# The Food Factor in Disease

**Published:** 1906-06

**Authors:** 


**The Food Factor in Disease.** By FRANCIS HARE, M.D. 2 vols. Pp. xiv., 497; viii., 535. London: Longmans, Green & Co. 1905.

The main argument of this book is to prove that many manifestations of disease are produced, or at any rate aggravated, by the overloading of the body with an excess of carbon-containing food. To such an excess of carbonaceous material in the blood the author gives the name of hyperpyræmia (Gr., πυρεια=fuel). Dividing the constituents of food stuffs into two main groups, the nitrogenous or proteid division and the carbonaceous, he maintains that the former corresponds to the building material and is adapted to the stimulation and repair of the functionating tissues, and indirectly therefore dominates the amount of carbonaceous material in the blood. The carbonaceous material, consisting of fats and carbo-hydrates, constitutes the fuel of the organism. The author maintains that the carbon contents of the blood are much more liable to variation than the nitrogenous, the variations being due to intermittence of food supply, fluctuations in the rate of combustion, and to intermenstrual accumulation. When an excess of carbonaceous material has been thus accumulated in the blood, the condition may be relieved physiologically by regulation of the income by means of the glycogenic distension of the liver, by increased combustion as by exercise, increased demand for bodily heat, by fat formation, and by menstruation, lactation, and utero-gestation. Pathologically the excess may be dispersed by many morbid processes, which include asthma, epilepsy, gout, &c. He maintains that of the fundamental food errors, excessive introgenous and deficient carbonaceous intake are probably unimportant, as they tend to be rapidly adjusted by the self-regulating power of the organism. Deficient introgenous intake and excessive carbonaceous intake are much more important, because they do not necessarily undergo such rapid automatic adjustment.

The pathological acarbonising processes are grouped like the physiological into divisions, *e.g.* (1) Those which regulate intake, viz. secondary dyspepsia, anorexia, bilious attacks, migraine, and gastralgic paroxysms. (2) Those which operate mainly through some form of increased expenditure; (*a*) by increased katabolism, *e.g.* epilepsy, asthma, angina; (*b*) by increased anabolism, as tumour formation; (*c*) by direct loss, of which glycosuria and some forms of hemorrhage may be examples. These pathological processes are to be regarded as variations, with modifications, of physiological mechanisms. While fully recognising the humoral condition of hyperpyræmia as the primary factor of many pathological acarbonising processes, he admits that in most cases the operation of numerous secondary factors is no less essential. Persistent high-blood pressure, arterio-sclerosis, and granular kidney he regards as due to hyperpyræmia, the uric acid and purin factor in disease being controlled by the hyperpyræmia. The author argues that uricæmia may be better controlled by diminishing the carbon-aceous elements in the blood and thereby facilitating greater uric acid excretion, than by diminishing the proteid intake and thereby simultaneously lowering the vitality of the body. A

large amount of clinical matter is introduced in support of the various hypotheses.

The work is one of very great interest, and contains much that is original in thought, although some of the hypotheses prove untenable. The book is one which should stimulate both clinical and pathological investigation along the lines suggested.

